# Prediction of 30-day pediatric unplanned hospitalizations using the Johns Hopkins Adjusted Clinical Groups risk adjustment system

**DOI:** 10.1371/journal.pone.0221233

**Published:** 2019-08-15

**Authors:** Mitchell G. Maltenfort, Yong Chen, Christopher B. Forrest

**Affiliations:** 1 Applied Clinical Research Center, Roberts Center for Pediatric Research, Children’s Hospital of Philadelphia, Philadelphia, Pennsylvania, United States of America; 2 Department of Biostatistics and Epidemiology, University of Pennsylvania, Philadelphia, Pennsylvania, United States of America; Medical University Graz, AUSTRIA

## Abstract

**Background:**

The Johns Hopkins ACG System is widely used to predict patient healthcare service use and costs. Most applications have focused on adult populations. In this study, we evaluated the use of the ACG software to predict pediatric unplanned hospital admission in a given month, based on the past year’s clinical information captured by electronic health records (EHRs).

**Methods and findings:**

EHR data from a multi-state pediatric integrated delivery system were obtained for 920,051 patients with at least one physician visit during January 2009 to December 2016. Over this interval an average of 0.36% of patients each month had an unplanned hospitalization. In a 70% training sample, we used the generalized linear mixed model (GLMM) to generate regression coefficients for demographic, clinical predictors derived from the ACG system, and prior year hospitalizations. Applying these coefficients to a 30% test sample to generate risk scores, we found that the area under the receiver operator characteristic curve (AUC) was 0.82. Omitting prior hospitalizations decreased the AUC from 0.82 to 0.80, and increased under-estimation of hospitalizations at the greater risk levels. Patients in the top 5% of risk scores accounted for 43% and the top 1% of risk scores accounted for 20% of all unplanned hospitalizations.

**Conclusions:**

A predictive model based on 12-months of demographic and clinical data using the ACG system has excellent predictive performance for 30-day pediatric unplanned hospitalization. This model may be useful in population health and care management applications targeting patients likely to be hospitalized. External validation at other institutions should be done to confirm our results.

## Introduction

About one-third of pediatric healthcare costs result from hospital admissions [1]. In 2012 the average costs for a pediatric hospitalization in the United States was $6,415 at a rate of 7,928 stays per 100,000 population aged 0–17 years, but this increased to $11,143, and decreased to 2,505 stays per 100,000 population, when neonatal stays were excluded [2]. Health systems that seek to reduce costs or admissions, either to improve efficiency or patient flow, often target patients at high risk of hospitalization. To develop and aim appropriate programs, risk assessment tools are needed that can accurately identify an at-risk population. Unfortunately, there are few pediatric-specific risk assessment tools that can be used to segment a population by its need for care management or other preventive services [3].

Certain types of hospitalizations are predictable because they are scheduled admissions for such indications as chemotherapy, surgery, and diagnostic tests. The majority, however, are unplanned and thus have some degree of associated preventability. Although there have been several studies on risk factors for pediatric readmission [4–10], there has been less attention given to developing predictive models for unplanned hospitalizations in populations of children and adolescents.

Our aim in this study was to develop a parsimonious risk model that used patient demographic, clinical data, and service use data over a one-year period to predict unplanned hospitalization (i.e., excluding admissions scheduled in advance of the admission date) in the next 30 days. Rather than developing a completely novel model, we built on the established Johns Hopkins ACG System’s clinical markers as the core of our modeling approach [11]. Prior studies have demonstrated that the ACG system is useful to classify pediatric populations but by levels of healthcare service use [12–14], but none has used this risk adjustment system to predict pediatric unplanned hospitalizations.

## Methods

### Data source and study sample

This study was done using Electronic Health Record (EHR) data for patients seen in the Children’s Hospital of Philadelphia (CHOP) health system. CHOP includes a large primary and specialty care outpatient network and a major inpatient facility that services a primary healthcare market in the states of Pennsylvania, New Jersey, and Delaware. Data were extracted from the CHOP EHR System (Epic) for visits in outpatient, emergency department, and inpatient settings for patients with at least one physician visit in any of these settings from January 2009 to December 2016. During the study period, 920,226 patients met these selection criteria. Applying a criterion that the children not already be hospitalized at the start of the reference month (see following) reduced the population to 920,051. The CHOP Institutional Review Board designated this study as not human subjects research.

### Unplanned hospitalization

Because a portion of pediatric hospitalizations are scheduled for such activities as inpatient chemotherapy administration, neurological testing, and surgery and thus are not preventable, we focused on those that were unplanned. These hospitalizations have been confirmed as real events, and not administrative artifacts, by ensuring that the site of care was an inpatient place of service in the CHOP hospital. Unplanned hospitalizations were those that were not flagged as elective hospitalizations in an Admission/Discharge/Transfer table in our database. Among all confirmed hospital admissions during the study period, 87% were unplanned.

### Predictors

Clinical variables were derived from the ACG system and included its DxPM score (a diagnosis-based probability estimate for patient risk of future healthcare use [3]), number of chronic conditions (0, 1, 2, 3+), and number of hospital dominant conditions (0, 1, 2+), the latter defined as a diagnosis associated with at least a 50% probability of hospitalization among patients of all ages within the coming year [15]. DxPM was categorized based on the percentile value for the cohort in the preceding year: 0–50% was the default, and other categories were 51–75%, 76–85%, 86–95%, 96–98%, and 99%. Demographic predictors were patient age, gender, race/ethnicity, and insurance type. Age was treated as a categorical variable, with the age of the patient’s first visit during the prior year divided into three-month blocks up to three years and one-year blocks afterward up to age 18. We used finer age stratifications in the first year of life because infancy holds the highest risk of hospitalization (excluding inpatient stays for birth). The insurance types were binary variables, defined as whether prior coverage of the patient was public insurance, private or self-pay. The number of unplanned hospitalizations in the past year was categorized as 0, 1, or 2+; because prior hospitalizations turned out to be a strong predictor and we were concerned about potential bias using hospitalizations to predict hospitalizations, we tested an alternative model omitting this predictor.

### Statistical analyses

We generated 84 epochs (12 months x 7 years) on a sliding window of 12 months of patient data across 2009–2016. Each successive window began and ended a month later than the preceding. For instance, the period January 2009 through December 2009 was used to predict a hospitalization occurring in January 2010, and so on. We split the study population into a 70% training sub-sample to develop the models and a 30% test sample to test model performance on a different set of patients.

Logistic regression was used to model the risk of a patient being hospitalized in the current month, excluding patients who were already in hospital, prior to the current month and extending into or past the current month. For this exclusion, we did not limit to planned or unplanned hospitalizations, or apply the other checks used to confirm unplanned hospitalizations.

As the outcome is a binary variable representing whether the patient had any admissions in a given month, this will necessarily drop hospitalizations that are readmissions that follow an admission earlier in that month. Similarly, our exclusion rule drops admissions that are readmissions for patients who are excluded due to an ongoing hospitalization as described above.

A generalized linear mixed model (GLMM) for prediction of risk of hospitalization in the current month was created using the demographic, clinical and prior hospitalizations as predictors and accounting for multiple measurements from the same patient using a patient-level random effect which described how the patients’ individual risk might vary from the overall population controlling for the predictors. To account for time-varying trends, we also included month of epoch (12 values, January through December) and its position in the sequence (a real-valued number scaling from 1 to 84). The GLMM was implemented in the statistical computer language R [16] using the lme4 package [17].

The GLMM was used to derive risk scores computed as the beta coefficients from the model derived from the training sample and applied to the covariates for patients in the testing sample. The scores were based only on the demographic, clinical and prior use coefficients, not on patient-based random effects or the time-based predictors added to the GLMM. Patient-based effects had to be dropped as the random effects would not be applicable to the test set or any new group of patients. Time-based predictors would not be relevant within a given epoch. This approach allowed us to classify patients by risk of future hospitalization within a given epoch using a consistent approach across all epochs.

Area under the curve (AUC), which estimates the probability that a hospitalized patient will outscore a non-hospitalized patient, was used to describe how well the model can discriminate among patients at different risk for hospitalization. As a model can behave better on a training set than on a new set of data, model optimism was defined as the difference between the AUC for the training set and the AUC for the test set [18]; some decline in AUC is expected, as the model can fit noise as well as real effects in the data, but a large decline would indicate that the model results may not be generalizable.

## Results

Table 1 shows the distribution of clinical, demographic and hospitalization variables among patients. Because age, prior hospitalizations in past year, and clinical variables can all be expected to change across time windows, the table shows the number and percentage of patients with at least one record in a given value. The table also shows the distribution of time windows (epochs) that includes a particular patient, and the distribution of patient parameters across these epochs. 53,091 or 5.72% of patients were represented in all 84 epochs, and the median number of epochs per patient was 24. Finally, the total number of unplanned hospitalizations (barring exclusions for patients already in hospital, as described above) for each epoch were tallied and used to estimate the overall rate of hospitalization in a given month across all epochs and within specific categories.

**Table 1:** Distribution of patients and demographic/clinical variables. Left column is by individual patient and whether they had at least one epoch (time window) with a given factor. Middle column is total number of epochs, treating the same patient in different epochs as different records. Monthly hospitalization rate, the rightmost column, is calculated from the total number of hospitalizations and the total number of epochs in a given category.

	Total Patients	Total Epochs	Monthly Hospitalizations
**Number unique patients**	920,051	31,078,325	111,027 (0.36%)
**Epochs/patient, median (IQR)**	24 (12–53)		
**Female**	441,194 (48.0%)	14,989,311 (48.2%)	51,400 (0.34%)
**Race/Ethnicity**			
**White, not Hispanic**	496,757 (54.0%)	17,055,391 (54.9%)	43,082 (0.25%)
**Asian, not Hispanic**	34,217 (3.7%)	1,049,866 (3.4%)	3,632 (0.35%)
**Black, not Hispanic**	213,426 (23.2%)	7,855,904 (25.3%)	47,772 (0.61%)
**Hispanic**	60,663 (6.6%)	1,923,758 (6.2%)	10,063 (0.52%)
**Other or Missing**	114,988 (12.5%)	3,193,406 (10.3%)	6,478 (0.20%)
**Age—at least one measurement in category**			
**0–2 months**	154,918 (16.8%)	1,805,098 (5.8%)	16,712 (0.93%)
**3–5 months**	129,613 (14.1%)	535,292 (1.7%)	4,201 (0.78%)
**6–8 months**	129,865 (14.1%)	539,222 (1.7%)	3,477 (0.64%)
**9–11 months**	124,634 (13.5%)	524,519 (1.7%)	3,068 (0.58%)
**12–14 months**	127,756 (13.9%)	556,312 (1.8%)	3,047 (0.55%)
**15–17 months**	119,852 (13.0%)	516,396 (1.7%)	2,758 (0.53%)
**18–20 months**	117,867 (12.8%)	520,659 (1.7%)	2,592 (0.50%)
**21–23 months**	88,023 (9.6%)	419,244 (1.3%)	2,395 (0.57%)
**24–26 months**	112,650 (12.2%)	564,230 (1.8%)	2,473 (0.44%)
**27–29 months**	83,135 (9.0%)	410,769 (1.3%)	2,143 (0.52%)
**30–32 months**	84,284 (9.2%)	444,058 (1.4%)	1,972 (0.44%)
**33–35 months**	75,961 (8.3%)	414,903 (1.3%)	1,869 (0.45%)
**3 years**	172,417 (18.7%)	1,801,224 (5.8%)	6,772 (0.38%)
**4 years**	168,869 (18.4%)	1,781,483 (5.7%)	5,220 (0.29%)
**5 years**	164,842 (17.9%)	1,739,647 (5.6%)	4,727 (0.27%)
**6 years**	157,851 (17.2%)	1,662,944 (5.4%)	3,915 (0.24%)
**7 years**	152,822 (16.6%)	1,618,320 (5.2%)	3,591 (0.22%)
**8 years**	147,975 (16.1%)	1,563,507 (5.0%)	3,236 (0.21%)
**9 years**	143,943 (15.6%)	1,522,192 (4.9%)	3,384 (0.22%)
**10 years**	140,552 (15.3%)	1,492,691 (4.8%)	3,264 (0.22%)
**11 years**	142,530 (15.5%)	1,528,433 (4.9%)	3,382 (0.22%)
**12 years**	137,116 (14.9%)	1,456,056 (4.7%)	3,559 (0.24%)
**13 years**	133,433 (14.5%)	1,422,585 (4.6%)	3,677 (0.26%)
**14 years**	130,579 (14.2%)	1,389,163 (4.5%)	3,989 (0.29%)
**15 years**	125,169 (13.6%)	1,338,371 (4.3%)	4,210 (0.31%)
**16 years**	117,957 (12.8%)	1,239,062 (4.0%)	4,121 (0.33%)
**17 years**	101,792 (11.1%)	1,070,513 (3.4%)	3,291 (0.31%)
**18+ years**	73,401 (8.0%)	1,201,432 (3.9%)	3,982 (0.33%)
**Chronic Conditions—at least one measurement in category**			
**None**	785,790 (85.4%)	18,688,252 (60.1%)	28,789 (0.15%)
**1**	435,297 (47.3%)	7,966,175 (25.6%)	31,001 (0.39%)
**2**	185,205 (20.1%)	2,595,284 (8.4%)	17,790 (0.69%)
**3+**	91,944 (10.0%)	1,828,614 (5.9%)	33,447 (1.83%)
**Hospital Dominant Conditions—at least one measurement in category**			
**None**	916,059 (99.6%)	30,604,006 (98.5%)	93,619 (0.31%)
**1**	27,612 (3.0%)	434,191 (1.4%)	14,335 (3.30%)
**2+**	5,199 (0.6%)	40,128 (0.1%)	3,073 (7.66%)
**DxPM bracket—at least one measurement in category**			
**0–50%**	723,396 (78.6%)	15,547,535 (50.0%)	28,544 (0.18%)
**51–75%**	512,104 (55.7%)	7,773,999 (25.0%)	19,570 (0.25%)
**76–85%**	209,909 (22.8%)	3,107,732 (10.0%)	14,711 (0.47%)
**86–95%**	169,522 (18.4%)	3,105,043 (10.0%)	20,401 (0.66%)
**96–99%**	63,262 (6.9%)	1,236,683 (4.0%)	19,065 (1.54%)
**Top 1%**	14,327 (1.6%)	307,333 (1.0%)	8,736 (2.84%)
**Insurance—at least one visit paying by**			
**Public Pay**	330,832 (36.0%)	11,151,012 (35.9%)	71,707 (0.64%)
**Self Pay**	51,345 (5.6%)	1,765,225 (5.7%)	6,050 (0.34%)
**Private Pay**	676,163 (73.5%)	23,260,666 (74.8%)	62,798 (0.27%)
**Unplanned hospitalizations in the prior year—at least one record with**			
**None**	902,595 (98.1%)	29,528,532 (95.0%)	64,114 (0.22%)
**1**	110,715 (12.0%)	1,278,318 (4.1%)	22,570 (1.77%)
**2+**	22,858 (2.5%)	271,475 (0.9%)	24,343 (8.97%)

The 84 epochs contained an average of 369,980 patients (SD 21,759), of whom an average of 1,322 (SD 132) or 0.36% (SD 0.04%) were hospitalized in the next month. There was some seasonal effect: the rates in December and January averaged 0.40%, while those in July averaged 0.31% (S1 Fig). There was also evidence of a long-term decline over time with monthly hospitalization rates declining from about 0.37% in 2009 to 0.33% in 2016 (S2 Fig). The declining rate was due to fairly constant hospitalization counts with an increasing size of the at-risk population. Because of these trends, the GLMM model across all epochs included a linear term for decline of hospitalization rate and a month-based factor for the seasonal variation.

The GLMM model coefficients with standard errors are shown in in Table 2, positive values reflecting increased risk of hospitalization. We found that prior hospitalizations had a large predictive value for new hospitalizations, so for comparison, we also show the GLMM coefficients for the alternative model fit without prior hospitalizations as a predictor. A striking factor is the ‘U-shaped’ estimate of the effect of age, decreasing with age for the first several years of life, and then increasing again at age 13. Also note the seasonal variation, where risk is higher in the winter and lower in the summer. Although the alternative model without prior hospitalizations does not perform as well as the main model (see following), there is little difference in parameters between model fits.

**Table 2:** Model summary. GLMM coefficients (log odds ratios) from the model are used to generate a score for identifying patients at higher risk for hospitalizations. Standard errors from the model are included for context and GLMM coefficients from the alternative model (excluding prior hospitalization) presented for comparison.

	GLMM coefficients	Std. Error	GLMM coefficient without prior hospitalization
**Female**	0.045	0.009	0.041
**Race**			
**White, not Hispanic**	0 (reference)		
**Asian, not Hispanic**	0.168	0.025	0.171
**Black, not Hispanic**	0.451	0.011	0.483
**Hispanic**	0.258	0.018	0.288
**Other or Missing**	-0.261	0.019	-0.263
**Age**			
**0–2 months**	1.622	0.025	1.806
**3–5 months**	1.281	0.030	1.431
**6–8 months**	1.076	0.031	1.205
**9–11 months**	0.961	0.032	1.078
**12–14 months**	0.925	0.032	1.034
**15–17 months**	0.857	0.033	0.954
**18–20 months**	0.800	0.033	0.885
**21–23 months**	0.824	0.034	0.906
**24–26 months**	0.706	0.034	0.781
**27–29 months**	0.721	0.035	0.789
**30–32 months**	0.632	0.036	0.696
**33–35 months**	0.635	0.036	0.686
**3 years**	0.517	0.027	0.556
**4 years**	0.280	0.028	0.298
**5 years**	0.208	0.028	0.215
**6 years**	0.043	0.030	0.041
**7 years**	-0.007	0.030	-0.013
**8 years**	-0.047	0.031	-0.054
**9 years**	0.015	0.030	0.011
**10 years**	0 (reference)		
**11 years**	0.024	0.030	0.028
**12 years**	0.081	0.030	0.084
**13 years**	0.126	0.030	0.137
**14 years**	0.229	0.030	0.243
**15 years**	0.287	0.029	0.308
**16 years**	0.320	0.030	0.347
**17 years**	0.176	0.032	0.200
**18+ years**	0.089	0.032	-0.141
**Chronic Conditions**			
**None**	0 (reference)		
**1**	0.647	0.012	0.675
**2**	0.941	0.015	1.004
**3+**	1.250	0.016	1.346
**Hospital Dominant Conditions**			
**None**	0 (reference)		
**1**	0.452	0.015	0.564
**2+**	0.683	0.029	0.890
**DxPM bracket**			
**0–50%**	0 (reference)		
**51–75%**	0.171	0.012	0.161
**76–85%**	0.191	0.015	0.183
**86–95%**	0.243	0.015	0.283
**96–99%**	0.472	0.018	0.507
**Top 1%**	0.730	0.023	0.760
**Insurance**			
**Public Pay**	0.440	0.010	0.447
**Self Pay**	-0.232	0.019	-0.270
**Unplanned Hospitalizations in the Prior Year**			
**None**	0 (reference)		
**1**	0.670	0.011	Omitted
**2+**	1.079	0.014	
**Monthly effect**			
**Jan**	0 (reference)	0.018	
**Feb**	-0.106	0.017	-0.106
**Mar**	0.029	0.018	0.030
**Apr**	-0.077	0.018	-0.075
**May**	-0.091	0.018	-0.089
**Jun**	-0.242	0.018	-0.239
**Jul**	-0.264	0.018	-0.262
**Aug**	-0.203	0.018	-0.201
**Sep**	-0.141	0.018	-0.138
**Oct**	-0.069	0.017	-0.066
**Nov**	-0.064	0.017	-0.061
**Dec**	-0.012	0.018	-0.009

Omitted from Table 2 for clarity are two parameters which are not included in the GLMM-derived score, although they are included in the calculation of predicted hospitalization risk for patients in a given epoch. One parameter is the intercept (baseline value), which for the main model is -7.381 (SE 0.027), corresponding to a baseline risk of hospitalization of 0.06% per month. The other is the per-epoch adjustment, which has a coefficient of -0.048 (SE 0.002) per year.

The fixed effects, without the time-dependent predictors per month or per epoch, were used to generate a score to identify hospitalization risk for patients within each epoch. The results were compared for the training and test patient populations. The AUC for all epochs was 0.826 in the training set and 0.821 in the test set, suggesting negligible overfitting. When we omitted prior hospitalizations, AUC fell to 0.808 for training and 0.802 for test. There were no visible trends in AUC over time.

Table 3 shows how the decile of calculated score compares to both the observed hospitalization rates and the predicted rates from the GLMM including time-varying fixed effects but not patient-level random effects. These random effects were left out of the prediction calculation because they are not available for the test set and will not be available for patient populations outside our own. The intra-class correlation coefficient for the GLMM is 0.215, indicating that 21.5% of the variability in results can be attributed to patient-specific factors that would be accounted for in the omitted patient-level random effect. Deciles were calculated within epoch so that it would be possible to get an idea of variability.

**Table 3:** Observed rates, predicted rates and observed/predicted ratios within deciles of scores. 30% test sample (separate from 70% training sample used to create GLMM) used. Deciles are calculated within each epoch so it is possible to get an idea of variability by calculating SD across epochs.

Decile	Observed %Hosp, Mean (SD)	Predicted %Hosp, Mean (SD)	Obs/Pred, Mean (SD)
**1**	0.05% (0.02%)	0.06% (0.01%)	0.86 (0.42)
**2**	0.06% (0.02%)	0.07% (0.01%)	0.94 (0.37)
**3**	0.08% (0.03%)	0.09% (0.01%)	0.98 (0.31)
**4**	0.10% (0.03%)	0.11% (0.01%)	0.93 (0.25)
**5**	0.12% (0.04%)	0.14% (0.02%)	0.86 (0.27)
**6**	0.15% (0.04%)	0.17% (0.02%)	0.92 (0.21)
**7**	0.21% (0.05%)	0.22% (0.02%)	0.97 (0.23)
**8**	0.31% (0.06%)	0.29% (0.03%)	1.05 (0.17)
**9**	0.49% (0.09%)	0.41% (0.05%)	1.18 (0.17)
**10**	1.97% (0.29%)	1.11% (0.14%)	1.78 (0.16)

**Fig 1:**
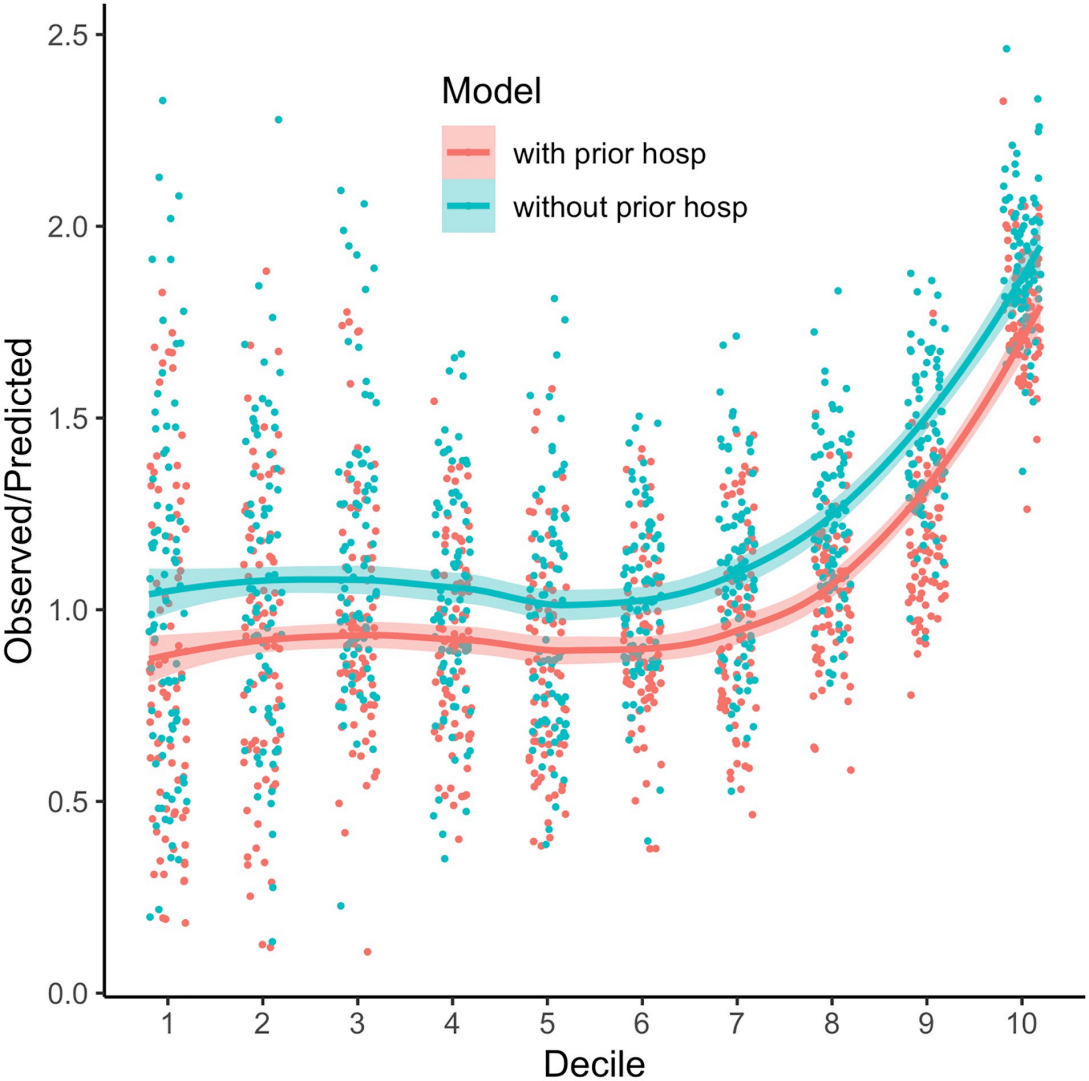
Ratio of observed/predicted for the main model (with prior hospitalization as a predictor) and the alternative (without prior hospitalization) plotted against decile for each score. 30% test sample (separate from 70% training sample used to create GLMM) used. Deciles and observed/predicted rates are calculated within each epoch to show potential variability.

Note that at the highest decile, the model prediction underestimates the true unplanned hospitalizations. Plotting the ratio of observed/predicted rates against decile (Fig 1), we see that the main model tends to under-estimate lower risks of hospitalization, and that the observed/predicted ratios have parallel increases with decile. Comparing the main model to the model without prior hospitalizations, we can see that the reduced model further under-estimates the percentage at higher rates. The higher AUC for the main model may be attributable to better discrimination between low- and high-risk patients, even if the actual assessment of risk is biased.

To examine the feasibility of targeting patients at greater risks of hospitalization, we looked at hospitalizations captured in groups defined by increasing cut-offs of score based on percentile within an epoch using data from the test sample. Using a 10% cut-off, an average of 56% of all observed unplanned hospitalizations were captured in the group of records above the cut-off, the top 5% accounted for 43% and the top 1% accounted for 20% of hospitalizations.

To address whether the model bias at higher rates could be attributed to specific diagnoses, we calculated the ratio of average hospitalizations and average predicted rate for each patient in the test set and linked the resulting table to the condition records to determine which Major Expanded Diagnosis Clusters (MEDC) from the ACG system were associated with higher ratios of observed hospitalization to predicted rates. We limited the analysis to those conditions associated with direct visits (inpatient, outpatient, ER or observation). The MEDC codes overlap with the ACG aggregate fields (hospital dominant conditions, chronic conditions, DxPM) [11] so this analysis would indicate which clinical findings may require additional weight in a predictive model.

## Discussion

This study sought to determine whether the Johns Hopkins ACG risk adjustment system is useful for the specific question of hospitalization risk within the limited population of pediatric patients. The results are encouraging. The AUC, describing discrimination power of the scoring model, is 0.821. The closest analogue in the literature to the current model may be predictive models for 30-day readmissions, and prior studies did not see an AUC above 0.83 and only a minority of studies had AUC above 0.70 [8, 19]. There are two benefits of this. One is that we have a new assessment of what risk factors hold for pediatric patients. Although some of our findings, such as the effect of race, may be more specific to our patient cohort, the seasonality and age-based coefficients may be of more general applicability. The other is that we have shown that an existing validated clinical software package can be used to distill a patient’s potentially complex history into a parsimonious set of predictors for outcome modeling.

For our model, we must consider whether further refinements could improve performance, particularly among the highest risk patients. One avenue for expanding the current model is in considering hospitalization risk beyond the current month. However, a model which predicts multiple hospitalizations over a period of a few months may require added sophistication to account for correlations between longitudinal measurements for the same patient. Tools for such models are currently available [20] but still relatively experimental.

An assumption of our model is that all prior admissions are equal, but we do not distinguish between admission and readmission or whether there are readmissions that would lead to more than one hospitalization in given month. The question of whether all admissions are the same may also impact the outcome being modeled. For example, Leyenaar et al considered whether the time-sensitive nature of some conditions made direct admission or admission through ER more appropriate for some patients [21].

It is reasonable to assume that patients at greater risk for short-term readmission may also be at increased risk for hospitalization over a longer time frame [22]. The type and extent of surgery is known to affect readmission rate [5, 7], as is length of stay during a hospitalization [14, 23]. Auger and Davis found that patients admitted on a weekend were more likely to be readmitted within 30 days [10]. All of these factors should be available in a database.

Cecil et al followed a birth cohort specifically to examine factors affecting unplanned admissions [24]. They found that higher usage of outpatient visits, indicating a sicker child, is a potential indicator of greater risk of unplanned admissions; among 5–9 year-old children, an additional sick outpatient visit per year increased the risk of unplanned admissions by 23%. The other finding of note from this study was that incomplete vaccinations increased the risk among 1–4 year-olds children by 89%. Outpatient visits are one indicator of children who are sicker or otherwise more prone to hospitalization. Another is emergency visits, which have been seen as a factor in hospitalization [25] and readmission [5] rates. These are examples of additional predictors that could be added to our model.

Our predictive model for unplanned hospitalization does not consider environmental factors such as climate, pollution, or family situation. These data are now readily available by linking EHR data to area-level data-sets using the patient’s residence and converting it to census block or tract [26]. The current effort was deliberately limited to information that would be available solely in EHRs.

## Supporting information

S1 Fig Seasonal dependence of hospitalization rate. Across the 84 epochs, the rate of hospitalization per epoch is plotted against month and a loess smoother used to estimate an average. Shaded region is 95% confidence interval. This curve agrees with expectation that cold weather carries greater health risks.
(TIFF)

S2 Fig Monthly hospitalization rate by consecutive epoch (time window). There is a clear decline with time of the hospitalization rates. This reflects a relatively constant number of hospitalizations while the number of patients in the population increases.
(TIFF)
