# Supplementary figures and images for: Prediction of 30-day pediatric unplanned hospitalizations using the Johns Hopkins Adjusted Clinical Groups risk adjustment system
Source: PLoS One. 2019 Aug 15;14(8):e0221233. doi: 10.1371/journal.pone.0221233 (PMC6695224; doi:10.1371/journal.pone.0221233)

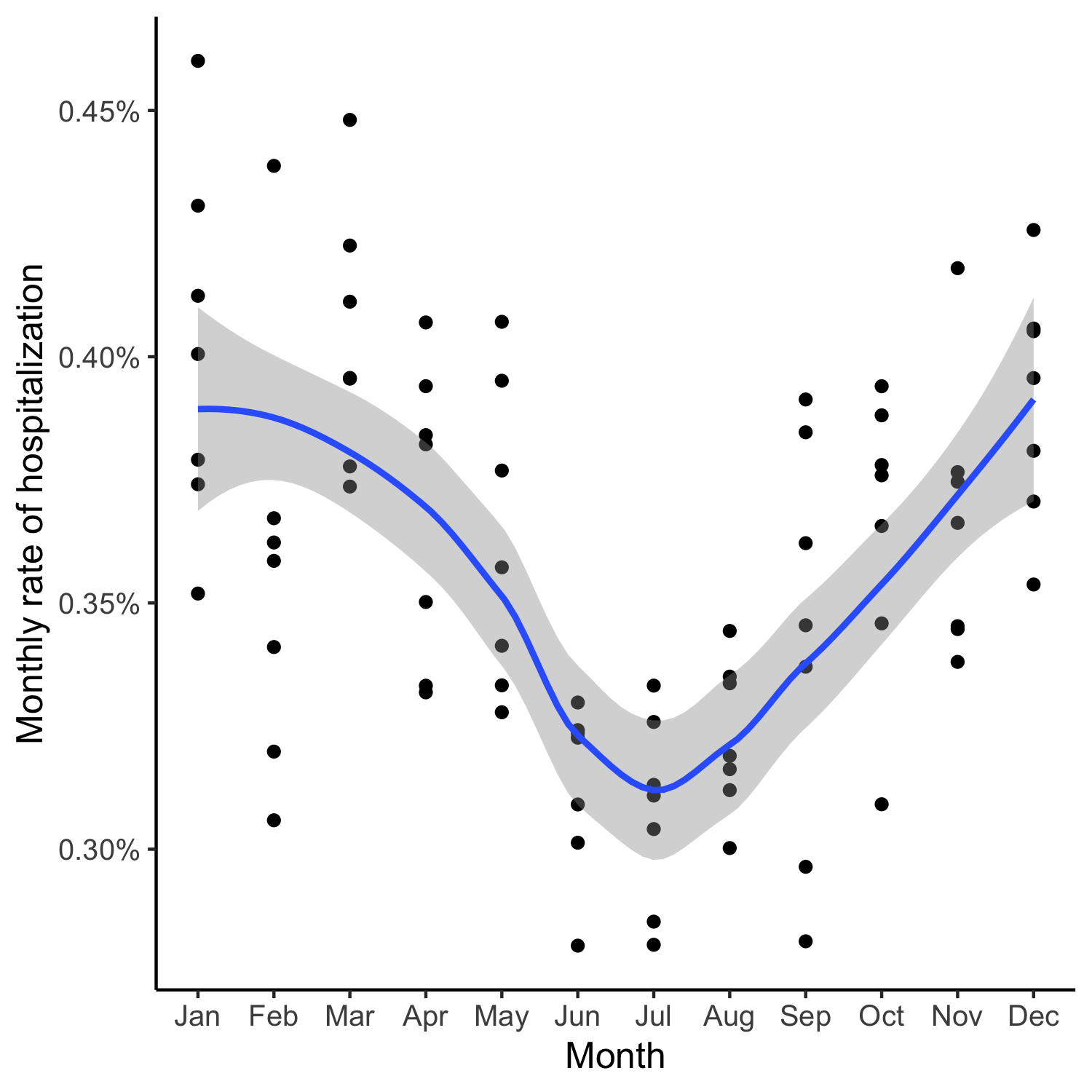

Supplement: S1 Fig — Across the 84 epochs, the rate of hospitalization per epoch is plotted against month and a loess smoother used to estimate an average. Shaded region is 95% confidence interval. This curve agrees with expectation that cold weather carries greater health risks. (TIFF) [file pone.0221233.s001.tiff]

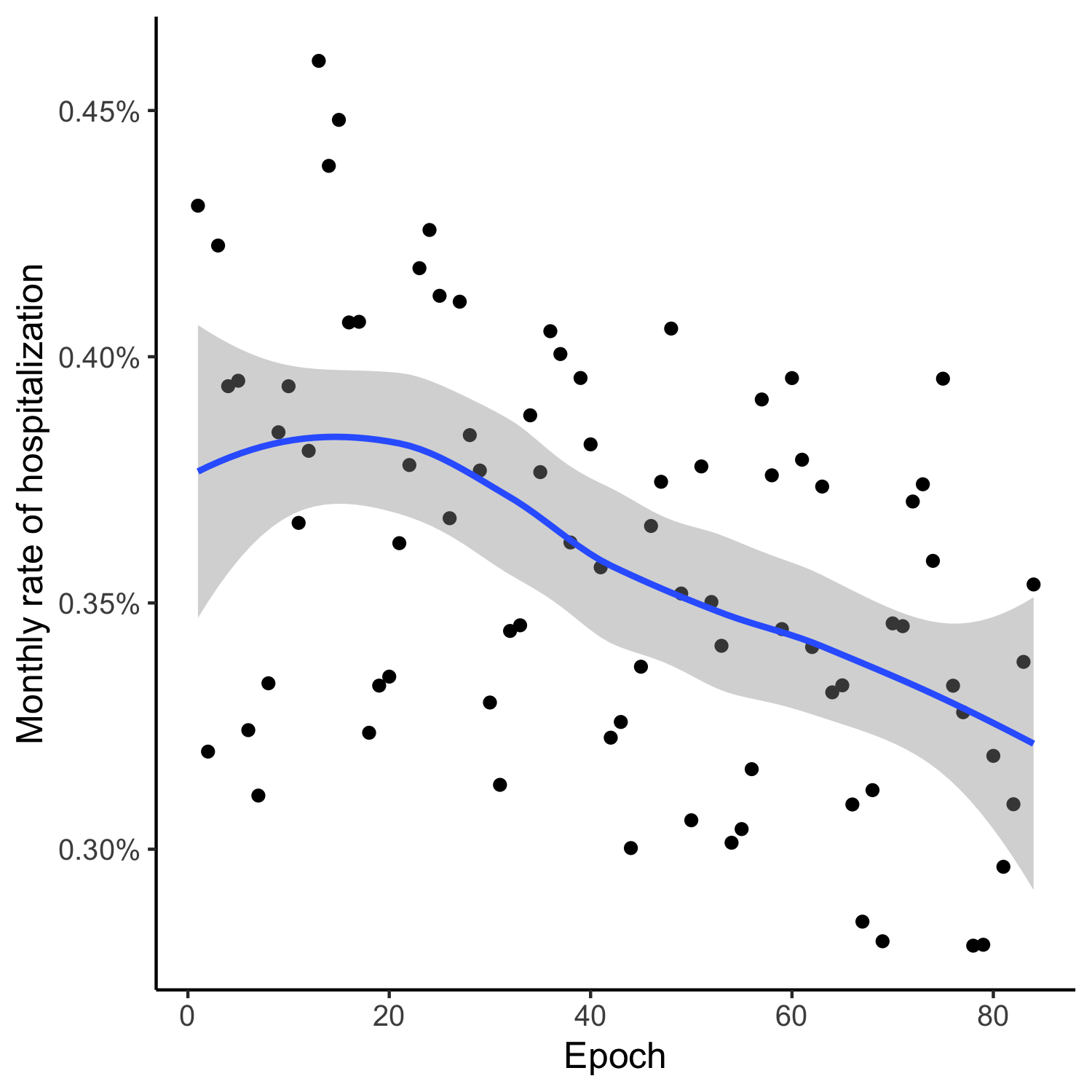

Supplement: S2 Fig — There is a clear decline with time of the hospitalization rates. This reflects a relatively constant number of hospitalizations while the number of patients in the population increases. (TIFF) [file pone.0221233.s002.tiff]
